# Vasoprotective effects of lysophosphatidic acid inhibit vascular injury caused by SARS-CoV-2 infection

**DOI:** 10.1038/s41598-025-06569-7

**Published:** 2025-07-25

**Authors:** Fumitaka Muramatsu, Naoi Hosoe, Tatsuya Suzuki, Teppei Shimamura, Yumiko Hayashi, Kazuhiro Takara, Lamri Lynda, Anna Shimizu, Weizhen Jia, Yoshimi Noda, Nobuyuki Takakura, Toru Okamoto, Hiroyasu Kidoya

**Affiliations:** 1 Department of Integrative Vascular Biology, Faculty of Medical Sciences, University of Fukui, 23- 3 Matsuoka-Shimoaizuki, Eiheiji, Yoshida, Fukui 910-1193 Japan; 2 Department of Signal Transduction, Research Institute for Microbial Diseases, Osaka University, Suita, Osaka 565-0871 Japan; 3 Department of Microbiology, Juntendo University School of Medicine, Tokyo, 113- 8421 Japan; 4 Department of Computational and Systems Biology, Medical Research Institute, Institute of Science Tokyo, Bunkyo-ku, Tokyo, 113-8510 Japan; 5 Integrated Frontier Research for Medical Science Division, Institute for Open and Transdisciplinary Research Initiatives, Osaka University, Suita, Osaka 565-0871 Japan

**Keywords:** Lysophosphatidic acid (LPA), COVID-19, Vascular injury, Vascular endothelial cell, SARS-CoV-2, Viral infection, Cardiovascular biology, Cell biology, Drug discovery, Microbiology, Molecular medicine

## Abstract

Vasculitis and vascular injury are induced in coronavirus disease 2019 (COVID-19) patients, suggesting an association between multi-organ failure and sequelae. Vascular endothelial dysfunction induced by severe acute respiratory syndrome-related coronavirus-2 (SARS-CoV-2) disrupts vascular barrier function, activates coagulation pathways, and induces extravasation of inflammatory cells. In addition to employing standard anti-inflammatory and antiviral agents, using treatments that stabilize and protect blood vessels following SARS-CoV-2 infection is a potentially effective strategy to relieve COVID-19 severity. Here, we focused on the vasoprotective effects of lysophosphatidic acid (LPA), a lipid mediator with diverse activities, and investigated whether it could be a novel therapeutic for COVID-19. Three-dimensional cultures of various human vascular endothelial cells that form luminal structures showed increased expression of LPA4 and entry receptors for SARS-CoV-2 infection, mimicking blood vessels in vivo. Validation using this culture system showed that LPA attenuated SARS-CoV-2 infection-induced vascular destruction and the activation of inflammatory signaling. In experiments with infected animals, LPA administration protected blood vessels and suppressed inflammation and vascular damage in lung tissue. The activation of LPA signaling in vascular endothelial cells may be an effective therapeutic approach for mitigating vascular injury in COVID-19.

**Supplementary Information:**

The online version contains supplementary material available at 10.1038/s41598-025-06569-7.

## Introduction

In addition to respiratory symptoms such as pneumonia, severe coronavirus disease 2019 (COVID-19) is associated with tissue damage attributed to a cytokine storm triggered by the systemic release of pro-inflammatory cytokines. Characteristically, vasculitis and vasculopathy have been reported to be induced, suggesting an association with multi-organ failure in COVID-19^1–3^. These symptoms are associated with the severity of COVID-19. In addition to these vascular disorders, vasculitis and vascular damage in the microvessels of systemic organs, such as the lungs and heart, may contribute to thrombus formation by activating coagulation^4^. The persistence of such microthrombi in systemic organs may cause various sequelae, including pulmonary fibrosis, neurological complications, and cardiovascular disorders, with medium- to long-term adverse effects^5^. Furthermore, the importance of protecting against vascular injury is evident from the fact that vascular dysfunction occurs under conditions of old age, obesity, hypertension, and diabetes mellitus, all of which are considered risk factors for COVID-19^6^.

Severe-acute-respiratory syndrome-related coronavirus-2 (SARS-CoV-2) has been suggested to infect vascular endothelial cells via angiotensin-converting enzyme 2 (ACE2) and Nrp1, which are expressed on the surface of host cells^7^. Evidence of ACE2 expression and replicative infection by SARS-CoV-2 in human endothelial cells is lacking^7,8^. Nevertheless, multiple studies have reported that vascular endothelial dysfunction can occur during SARS-CoV-2 infection through direct and indirect mechanisms, including inflammatory cytokine effects^9,10^. Vascular endothelial cells form tubular structures in the lumen of blood vessels and exert their barrier function by controlling vascular permeability, maintaining appropriate cell-to-cell associations via adhesion molecules such as VE-cadherin. Vascular endothelial dysfunction during SARS-CoV-2 infection causes endothelial cell disruption, lysis, and death, leading to endothelial inflammation^11^. As this occurs, vascular barrier function is disrupted, resulting in tissue edema due to increased vascular permeability. The coagulation pathway is activated by the expression of P-selectin, von Willebrand factor, and fibrinogen^12^. Moreover, increased expression of leukocyte adhesion molecules in vascular endothelial cells leads to dysregulation of inflammatory cell infiltration, such as enhanced extravasation of neutrophils and other cells.

Lysophosphatidic acid (LPA) is a lipid mediator with various activities, such as cell proliferation, differentiation, and migration, and is involved in various biological phenomena, such as central nervous system development, blood vessel formation, and wound healing. LPA is biosynthesized by the hydrolysis of lysophosphatidine choline by autotaxin^13^. Mice lacking the autotaxin gene have abnormal blood vessel formation during the fetal stage, resulting in lethality around embryonic day 10.5^14,15^. LPA signaling is activated by six subtypes of G protein-coupled transmembrane receptors: LPA1, LPA2, LPA3, LPA4, LPA5, and LPA6 and has an important role in angiogenesis^16,17^. We previously reported that the activation of LPA receptor 4 (LPA4) signaling in vascular endothelial cells induces the functional maturation of tumor vessels and improves drug delivery by enhancing VE-cadherin-mediated endothelial cell junctions^18^. Furthermore, using an orthotopic tumor transplantation model of brain tumors, it was clarified that the induction of vascular maturation by LPA improves the efficacy of immune checkpoint inhibitor therapy by improving immune cell infiltration into tumor tissue^19^. Few molecules are capable of vascular stabilization comparable to that exhibited by LPA, suggesting the potential of this unique molecule to be leveraged for treating conditions associated with vascular disorders. Especially, LPA is expected to be effective in the treatment of diseases characterized by pronounced vascular damage such as COVID-19. Hence, development of new LPA-based treatment modalities that differ from current antiviral and anti-inflammatory therapies is an intriguing area of research.

Here, we aimed to test the therapeutic efficacy of LPA on vascular injury and tissue damage seen in COVID-19. We analyzed pulmonary microvascular endothelial cells (HMVEC-Lung), brain microvascular endothelial cells (HMBECs), and renal glomerular endothelial cells (HRGECs) and conducted animal experiments using hamsters.

## Results

### Establishment of a 3D culture system for vascular endothelial cells

**Fig. 1:**
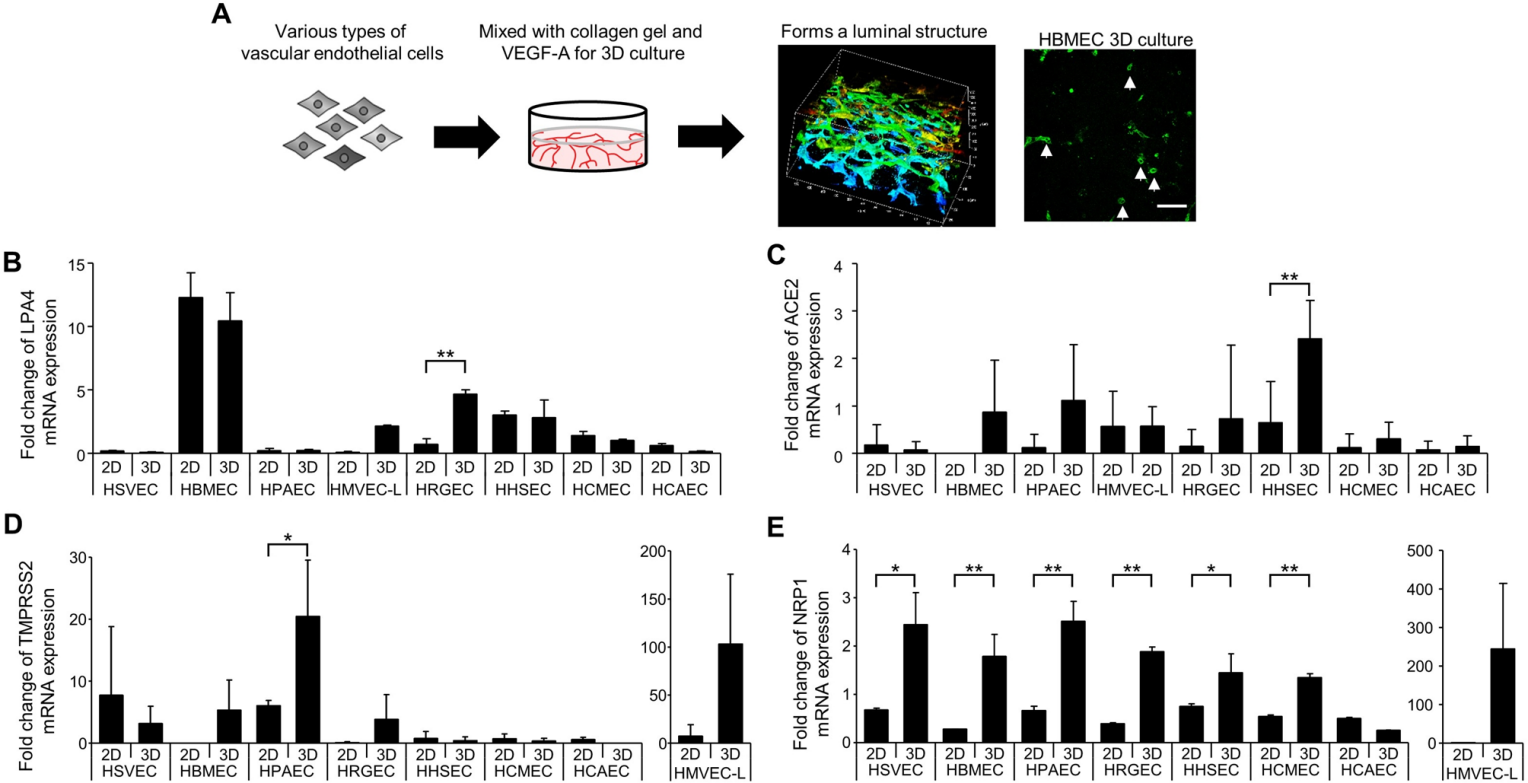
Expression of the genes for the LPA receptor, LPA4 and entry receptors for SARS-CoV-2 infection in various types of vascular endothelial cells. (**A**) Schematic diagram and image showing the formation of luminal structures in 3D cultures of vascular endothelial cells. The endothelial cells are stained with CD31 antibody, and the right image shows formed luminal structures of 3D-cultured HBMECs. Arrows indicate luminal structures. Scale bar, 100 μm. VEGF-A: vascular endothelial growth factor A. (**B**) Quantitative analysis of the mRNA expression of LPA4, a transmembrane receptor for LPA, in vascular endothelial cells cultured in planar culture (2D) or in three-dimensional culture in collagen gel (3D). The data are expressed as mean ± SD, n = 3. **P< 0.01 (**C–E**) Quantitative analysis of the mRNA expression of ACE2, TMPRSS2, and Nrp1, entry receptors for SARS-CoV-2 infection, in vascular endothelial cells cultured in planar culture (2D) or in three-dimensional culture in collagen gel (3D). The data are expressed as mean ± SD, n = 3. *P < 0.05, **P < 0.01.

To analyze vascular injury, it is necessary to create 3D cultures of vascular endothelial cells that form structures resembling in vivo lumens. An in vitro vascular analysis system was established to investigate whether LPA protects against vascular damage caused by SARS-CoV-2 infection. Primary human intravascular cell lines were derived from the lung, heart, brain, kidney, liver, and peripheral tissues, where tissue damage occurs during COVID-19 (Table 1). Vascular endothelial cells were cultured in collagen gels at appropriate concentrations to form luminal vessels. The 3D luminal structure of blood vessels began to form 24 h after culture establishment and persisted for 72 h, as demonstrated by the CD31-positive luminal structures in Fig. 1A. In lumen-forming vascular endothelial cells, LPA4, a transmembrane receptor for LPA, was expressed in HMVEC-Lung, HMBEC, and HRGEC lines (Fig. 1B). In addition, the expression of ACE2 and TMPRSS2, entry receptors for SARS-CoV-2 infection, was significantly elevated when the endothelial cells were cultured in 3D to form luminal structures versus 2D planar cultures (Fig. 1C, D, E). Based on the criteria of high LPA4 and SARS-CoV-2 receptor expression (Table 2), we proceeded with SARS-CoV-2 infection experiments using three types of vascular endothelial cells: HMBECs, HMVEC-Lung, and HRGECs. However, during extended culture experiments, HMVEC-Lung tubes tended to collapse, making them unsuitable for long-term infection studies (Supplemental Fig. 1).

**Table 1:** List of vascular endothelial cells used in this study.

	Origin of blood vessels	Distributor
HCAEC	Coronary artery	Lonza Group AG
HMVEC-L	Lung microvessel	Lonza Group AG
HPAEC	Pulmonary artery	Lonza Group AG
HCMEC	Cardiac microvessel	ScienCell Research Laboratories
HHSEC	Hepatic sinusoidal vessel	ScienCell Research Laboratories
HRGEC	Renal glomerular vessel	ScienCell Research Laboratories
HBMEC	Brain microvessel	ScienCell Research Laboratories
HSaVEC-c	Saphenous vein	PromoCell GmbH

**Table 2:** Expression levels of key receptors in different endothelial cell types.

	LPA4	ACE2	TMPRSS2	Selected for further study
HCAEC	Low	Low	Low	No
HMVEC-L	High	High	High	Yes
HPAEC	Low	High	Low	No
HCMEC	Low	Low	Low	No
HHSEC	Low	Moderate	Low	No
HRGEC	High	High	Low	Yes
HBMEC	High	Moderate	Moderate	Yes
HSaVEC-c	Low	Low	Low	No

### Vasoprotective effects of LPA

**Fig. 2:**
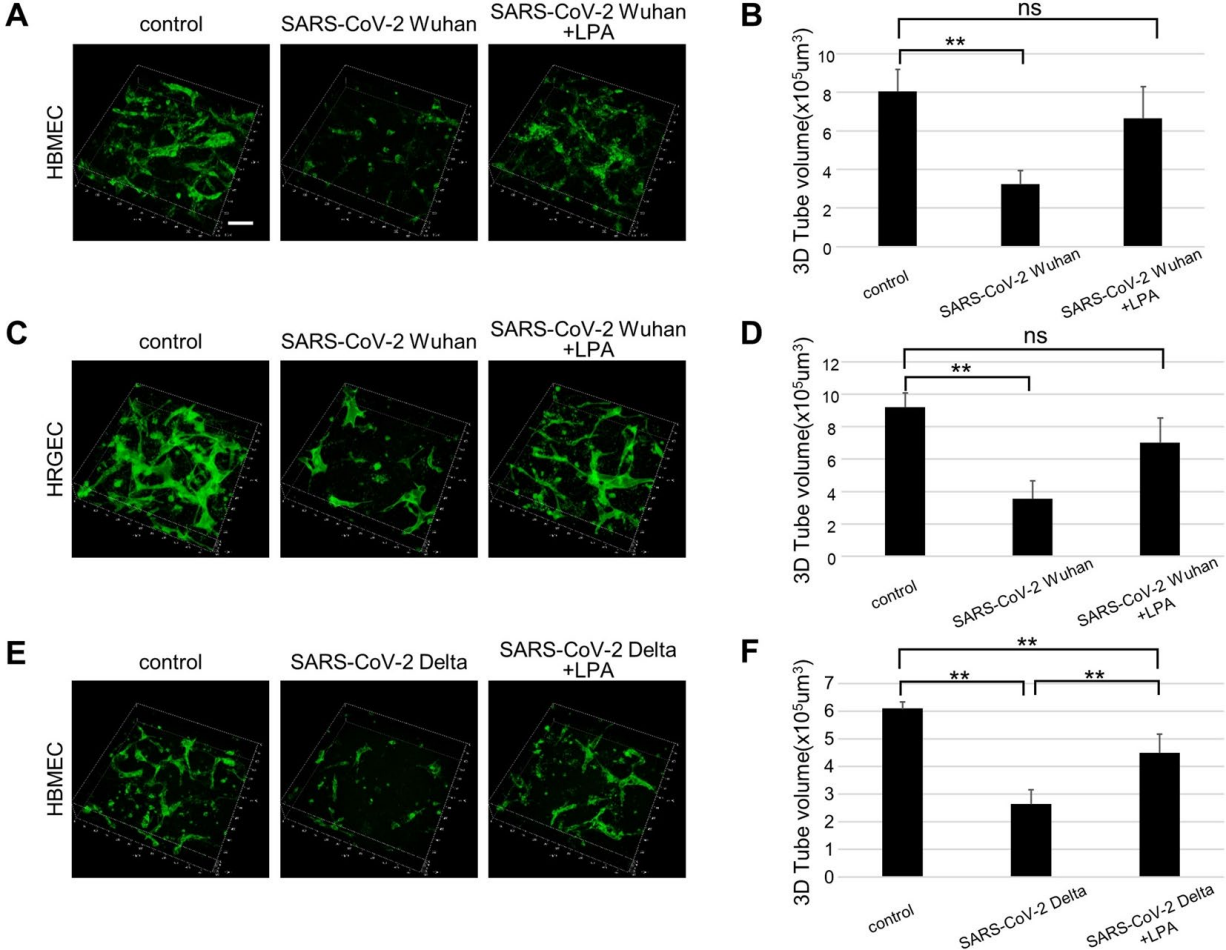
Analysis of vascular structure in a 3D human vascular culture system with SARS-CoV-2 infection and LPA treatment. (**A, B**) Representative image data of CD31-immunostained 3D-cultured HBMEC infected with SARS-CoV-2 Wuhan strain and the LPA-treated group. Pictures were taken from the bottom of the culture dish to 120 μm. The bar graph shows the measured volume of the 3D-cultured endothelial cell tube structure within a microscopic area. (**C, D**) 3D-cultured HRGEC infected with SARS-CoV-2 Wuhan strain and the LPA-treated group. (**E, F**) 3D-cultured HBMEC infected with Delta variant of SARS-CoV-2. The bar graph shows the measured volume of CD31 fluorescently labeled endothelial cell tube structures contained in the same amount of gel. All data are expressed as mean ± SD, n = 4 per group.**P < 0.01. ns, not significant. Scale bar, 100 μm.

The HMBEC and HRGEC lines were infected with the SARS-CoV-2 Wuhan strain at a multiplicity of infection of 0.1 after lumen construction by 3D culture on collagen gels. The infection caused disruption of the vascular structure, indicating that vascular damage had occurred. In contrast, the disruption of the vascular structure caused by SARS-CoV-2 infection was suppressed in the group treated with LPA, with only a slight decrease in tubular volume. In addition, the tubular volume of luminal vessels formed by HMBECs and HRGECs was significantly lower after SARS-CoV-2 infection relative to pre-infection volume (Fig. 2A-D). Similarly, infection with the Delta variant of SARS-CoV-2 caused the collapse of 3D HMBEC vessels, which was attenuated via LPA treatment (Fig. 2E, F). While HMVEC-Lung cells showed similar trends, their responses exhibited greater variability, producing less definitive results than those for HMBECs and HRGECs. Therefore, we focused our analysis on the latter two cell types. These results indicate that LPA treatment is effective in reducing vascular damage caused by SARS-CoV-2 infection by protecting blood vessels.

### Downregulation of pro-inflammatory genes by LPA

**Fig. 3:**
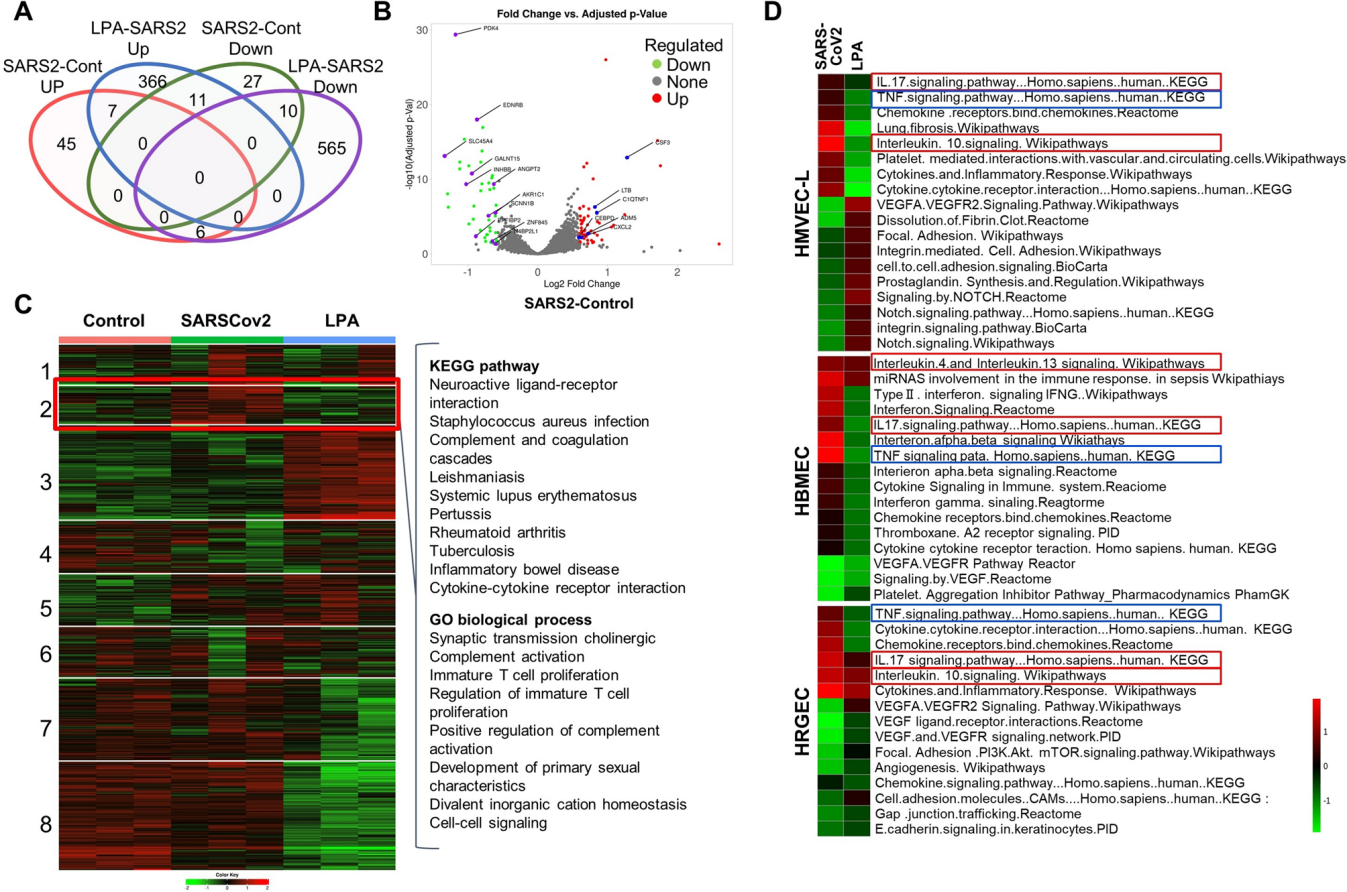
Gene expression analysis of 3D-cultured vascular endothelial cells following SARS-CoV-2 infection and LPA treatment. (**A**) Venn diagram of differentially expressed genes (DEGs) in vascular endothelial cells (HMVEC-Lung), comparing SARS-CoV-2 infection with control treatment (Cont) and LPA treatment with SARS-CoV-2 infection (FDR P < 0.05, fold change < 1.5). (**B**) Volcano plot showing upregulated (red) and downregulated (green) DEGs upon SARS-CoV-2 infection (left) and LPA treatment (right) in HMVEC-Lung. Purple spots indicate upregulated genes, and blue spots indicate downregulated genes when comparing SARS-CoV-2 with the control. (**C**) The k-means clustering analysis revealed 8 clusters in the 2,000 most variable genes in HMVEC-L. Pathway analysis of 116 genes in cluster 2 referred to GO biological processes and KEGG pathways. Transcriptional changes are heatmapped from green (strongly downregulated) to red (highly upregulated). Permission has been obtained from Kanehisa laboratories for using KEGG pathway database^42^. (**D**) Pathway analysis using KEGG database. A heatmap showing the activation of significantly enriched pathways, comparing SARS-CoV-2 infection with LPA treatment in HMVEC-Lung, HBMEC, and HRGEC. Pathways related to TNF signaling (highlighted in blue) and IL-17 signaling (highlighted in red) are shown. Enrichment values [-log(p-value)] are scaled from 0 to 1 (green to red). Permission has been obtained from Kanehisa laboratories for using KEGG pathway database^42^.

We analyzed gene expression to determine whether the activation of LPA signaling in vascular endothelial cells could suppress the inflammatory signaling pathways induced by SARS-CoV-2 infection. We conducted bulk RNA sequencing (RNA-seq) on HMBEC, HMVEC-Lung, and HRGEC, comparing SARS-CoV-2 infection with LPA treatment. The RNA-seq data on gene expression in HMVEC-Lung, comparing SARS-CoV-2 infection with the control, LPA treatment, are presented in Venn diagrams. The genes that were upregulated by more than 1.5 fold and downregulated by less than 1.5 fold (FDR < 0.05) are summarized in Fig. 3A. Of the 58 genes upregulated by infection, 6 were downregulated by LPA treatment; conversely, of the 48 genes downregulated by infection, 11 were upregulated by LPA treatment. Figure 3B shows a volcano plot of the specific genes. Transcript read counts for 15,156 genes in HMVEC-Lung were analyzed using k-means clustering of the 2,000 most variable genes (Fig. 3C). Of the 8 clusters, cluster 2 exhibited uniform transcriptional changes across 3 samples, with genes upregulated by SARS-CoV-2 infection and downregulated by LPA treatment. Based on the differentially expressed genes (DEGs), inflammation-related signals were examined using pathway analysis. The 116 genes in cluster 2 were mapped to Gene Ontology biological processes related to complement activation and immune reactions, and the associated KEGG pathways involved in infection and inflammatory signaling pathways were identified. TNF and IFN signals were activated by SARS-CoV-2 infection, but treatment with LPA effectively suppressed these signals (Fig. 3D). In addition, SARS-CoV-2 infection decreased the expression of genes related to cell adhesion, but their expression was restored by LPA treatment. These results were similar for HMBEC, HMVEC-Lung, and HRGEC. Overall, these gene expression analyses indicate that SARS-CoV-2 inoculation of 3D endothelial cell cultures results in upregulation of several key pro-inflammatory signaling pathways and downregulation of important cell adhesion genes, while LPA treatment downregulates these pro-inflammatory signaling pathways and restores the expression of cell adhesion genes.

### Therapeutic effects of LPA in an animal model

**Fig. 4:**
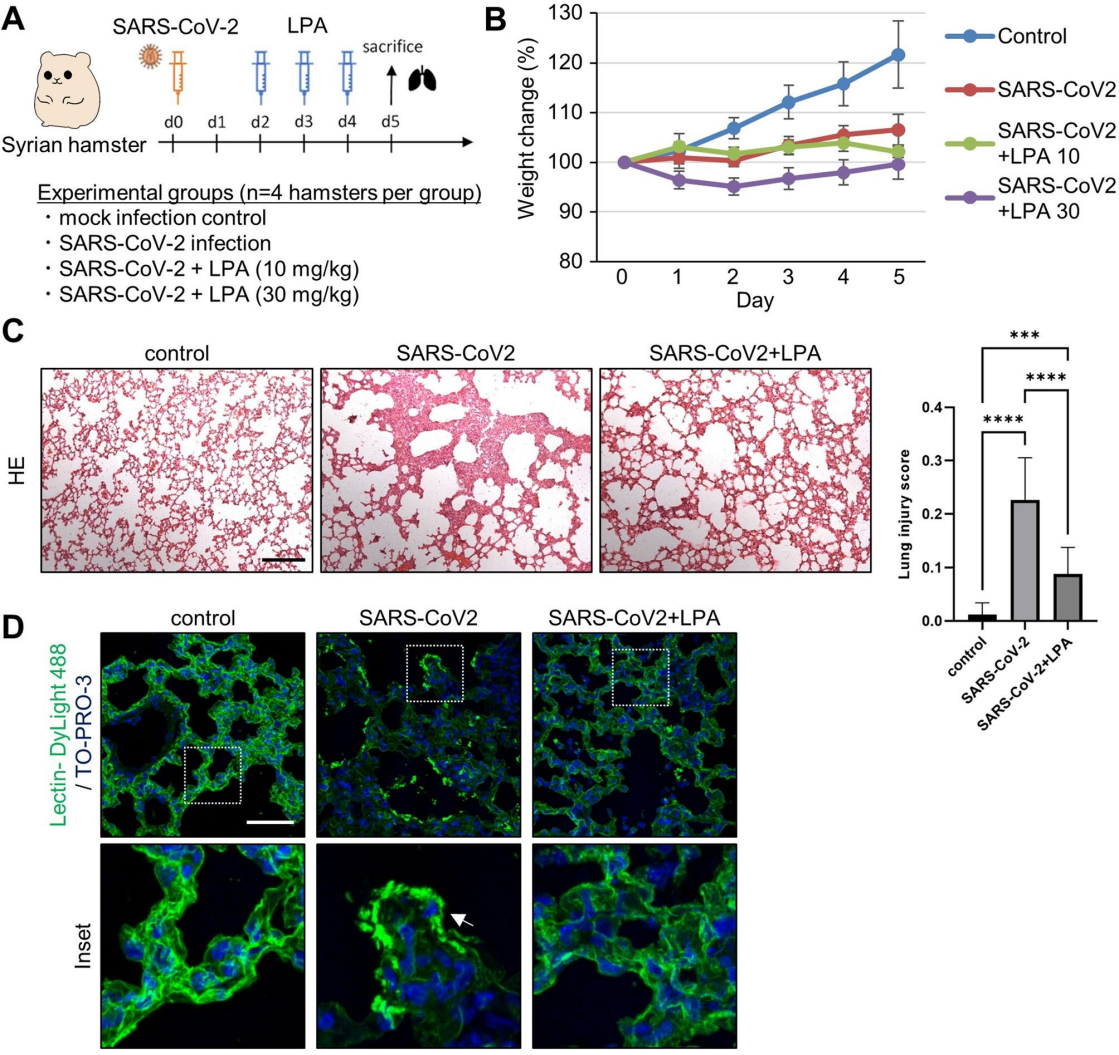
Therapeutic efficacy of LPA in a SARS-CoV-2-infected hamster model. (**A**) Schematic diagram of the timeline of LPA administration and analysis in a model of SARS-CoV-2 infection using Syrian hamsters. The diagram shows experimental groups, including the mock infection control, SARS-CoV-2 infection, SARS-CoV-2 + LPA (10 mg/kg), and SARS-CoV-2 + LPA (30 mg/kg), with n = 4 hamsters per group. (**B**) Percent weight change from baseline in Syrian hamsters over time after SARS-CoV-2 infection with and without LPA administration. The data are expressed as mean ± SD, n = 4 hamsters per group. (**C**) Representative images of hematoxylin and eosin staining of lung tissue harvested 5 d after SARS-CoV-2 infection with or without LPA (30 mg/kg). Semi-quantitative lung injury scores were graded based on injury criteria described in the Materials and Methods. Data are expressed as mean ± SD, n = 20. ****P< 0.0001. ***P = 0.0002. Scale bar, 200 μm. (**D**) Representative images of vascular staining with CD31 (green) and TO-PRO-3 (blue) of lung tissue harvested 5 d after SARS-CoV-2 infection with or without LPA (30 mg/kg). Arrows in the SARS-CoV-2 panel indicate areas where endothelial cells have detached from the basement membrane, creating gaps in the vascular lining and resulting in a disorganized vascular structure compared to the continuous endothelial lining seen in control and LPA-treated tissues. Scale bar, 100 μm.

The therapeutic vasoprotective effects of LPA against COVID-19 were examined in an animal model. SARS-CoV-2 infection was confirmed by the development of characteristic respiratory symptoms and pulmonary pathology in infected animals, consistent with previously established infection models^20,21^. Daily doses of LPA (10 or 30 mg/kg body weight) were administered intraperitoneally to Syrian hamsters (*n* = 4 per group) that were intranasally infected with SARS-CoV-2 (1.0 × 10^6^ PFU). Animals were housed under standard conditions (22 ± 2 °C, 12 h light/dark cycle, standard laboratory diet, and water *ad libitum*, housed in individually ventilated cages) and were euthanized by isoflurane overdose followed by cervical dislocation on day 5 after infection. (Fig. 4A). SARS-CoV-2 infection caused weight loss in hamsters, but LPA treatment did not inhibit this reduction in body weight (Fig. 4B). Lung tissue sections collected 5 days after infection were examined for inflammatory lesions by hematoxylin and eosin staining, and the tissue damage induced by SARS-CoV-2 infection was suppressed by LPA (Fig. 4C). Quantitative histopathological scoring revealed significantly reduced lung injury scores in LPA-treated groups compared to those in the SARS-CoV-2 infection group (*P* < 0.001), confirming the protective effect of LPA treatment. Furthermore, histological analysis of blood vessels in the lung tissue showed that vascular endothelial cells in the SARS-CoV-2-infected group were detached, as indicated by arrows in the high-magnification images (Fig. 4C), and the vascular structure was disorganized, whereas LPA administration improved vascular damage. These results suggest that LPA suppresses lung inflammation by inhibiting vascular injury caused by SARS-CoV-2 in an animal model.

### Expression of inflammation-related genes in infected tissues

**Fig. 5:**
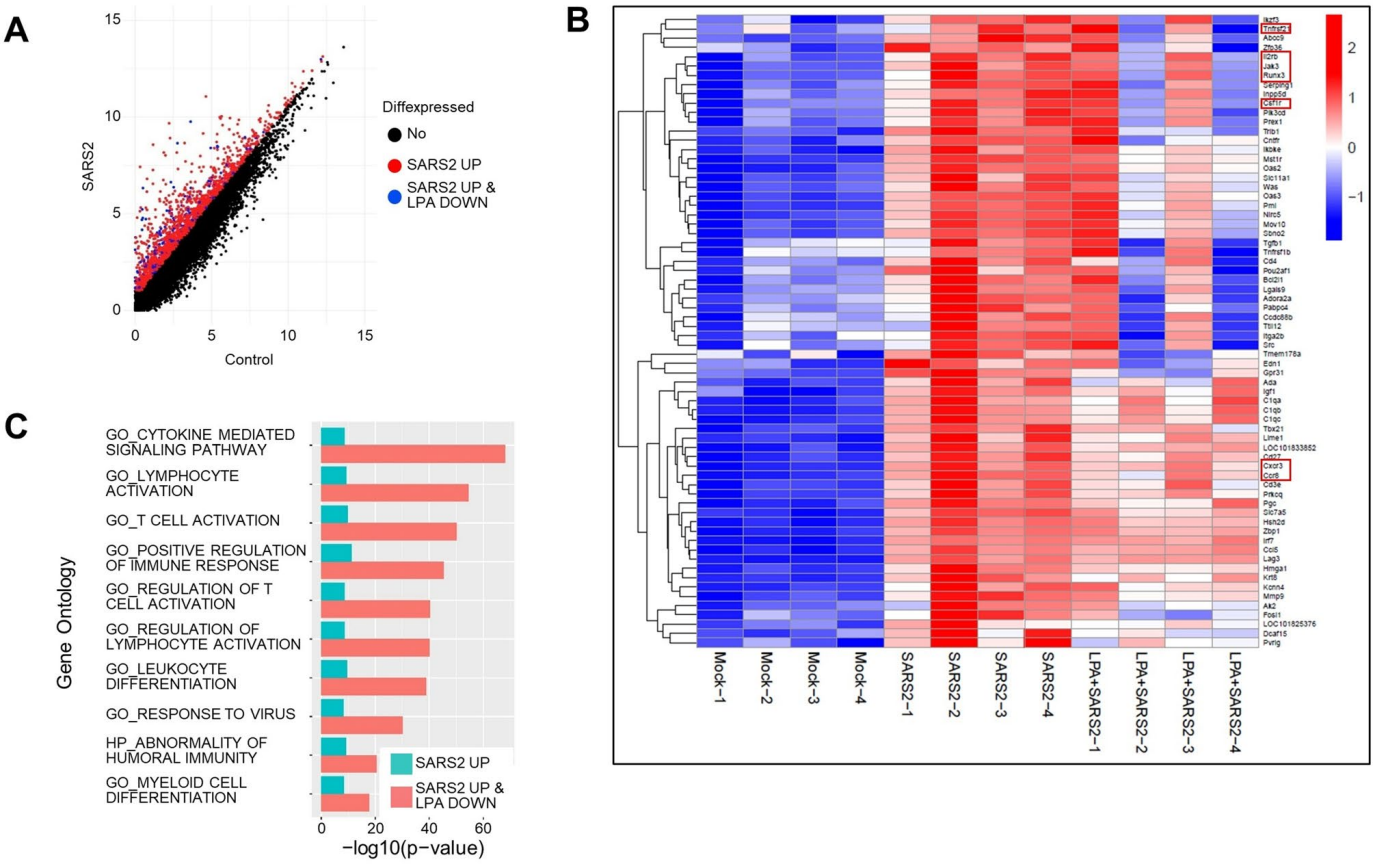
Evaluation of therapeutic efficacy of LPA by transcriptome analysis of lung tissue from hamsters challenged with SARS-CoV-2. (**A**) Dot plot showing DEGs in lung tissues from control vs. SARS-CoV-2-infected hamsters. RNA-seq of lung tissue harvested 5 d after SARS-CoV-2 infection reveals the upregulation of multiple genes (red dots), including several pro-inflammatory cytokines, while LPA administration results in the downregulation of a subset of pro-inflammatory genes (blue dots). (**B**) Heatmap showing gene expression patterns across experimental groups. Pro-inflammatory genes that were upregulated by SARS-CoV-2 infection and suppressed by LPA treatment are highlighted in red boxes. (**C**) Gene Ontology analysis visualization showing the activation of significantly enriched inflammatory pathways in lung tissue, comparing mock-infected controls, SARS-CoV-2-infected hamsters, and SARS-CoV-2-infected hamsters treated with LPA. Key inflammatory pathways affected by LPA treatment are highlighted. Permission has been obtained from Kanehisa laboratories for using KEGG pathway database^42^.

Comprehensive gene expression analysis by RNA-seq was performed on the lung tissue 5 days after infection with SARS-CoV-2. The results showed that SARS-CoV-2 infection induced the expression of genes for pro-thrombotic regulators of hemostasis, such as von Willebrand factor, key pro-inflammatory cytokines implicated in the cytokine storm, and endothelial cell adhesion molecules that promote leukocyte migration, such as Vcam1 and Icam1. In contrast, LPA treatment was associated with decreased expression of these pro-thrombotic and pro-inflammatory genes. (Fig. 5A, B). Additionally, Gene Ontology analysis showed that the upregulation of pro-inflammatory genes in lung tissue was suppressed following LPA administration (Fig. 5C). Taken together, these results suggest that LPA may be an effective therapeutic for suppressing vascular injury associated with severe COVID-19 disease.

## Discussion

In this study, we focused on the vascular stabilizing effects of LPA and examined whether it constitutes a novel therapeutic for COVID-19. We previously reported that LPA induces functional maturation of the tumor vasculature by inducing stabilization^18,19^. Tumor vessels are constantly exposed to an inflammatory environment that affects endothelial cells. Consequently, the junctions between the vascular endothelial cells are disrupted, leading to high leakage and reduced blood flow. The activation of LPA signaling enhances vascular endothelial cell adhesion by maintaining VE-cadherin on the plasma membrane and inhibiting vascular leakage. Analysis of gene-deficient mice showed that the induction of vascular stabilization by LPA is mediated by the LPA4 receptor. Although many molecules that activate vascular endothelial cells have been identified, molecules like LPA that stabilize blood vessels and protect them from inflammation are uncommon. The results of our gene expression and histopathological analyses suggest that LPA treatment preserves vascular integrity during SARS-CoV-2 infection, likely through the suppression of inflammatory signaling cascades. Administration of LPA did not prevent weight loss in our hamster infection model, possibly due to side effects caused by the action of LPA on cells other than vascular endothelial cells^22^. Therefore, an agonist that selectively acts on the LPA4 receptor is necessary to address LPA signaling as a therapeutic target for COVID-19. The vasoprotective effect of LPA on pulmonary inflammation in infected animal models suggests that LPA may be useful in suppressing the vascular injury observed in the tissues of COVID-19 patients and is expected to be a potential therapeutic agent.

COVID-19 is known to cause respiratory symptoms such as pneumonia and tissue damage due to systemic thrombus formation and cytokine storms. Vascular endothelial cell injury and inflammation have been implicated in the pathogenesis of these conditions^1,3,23^. Therefore, there is a need to develop therapeutic agents that inhibit not only viral proliferation but also vascular endothelial cell damage. LPA protects against SARS-CoV-2 infection-induced vascular damage, and its use in combination with antiviral drugs is expected to improve its therapeutic efficacy. In addition, many viruses that cause respiratory infections often have lingering effects, such as asthma, even after the virus has been cleared from the body, and there are concerns about the aftermath of COVID-19^24,25^. COVID-19 has been reported to cause significant vascular inflammation, leading to thrombus formation in the microvessels of the lungs, heart, and other systemic organs. It has been suggested that the persistence of such clots in systemic organs may have medium- to long-term adverse effects. Recent studies have further elucidated the mechanisms of vascular injury in COVID-19, confirming the central role of endothelial dysfunction in both acute infection and post-COVID syndrome^26,27^. These findings suggest that LPA may be particularly valuable for reducing the incidence of sequelae by inhibiting thrombus-related factors and suppressing inflammatory responses.

Gene expression and the properties of vascular endothelial cells differ between planar cultures for forming cell layers and 3D cultures for forming luminal structures^28^. To analyze the vascular injury caused by COVID-19 in vitro under conditions similar to those found in vivo, vascular endothelial cells that have formed a luminal structure are required. In this study, we constructed a 3D culture system capable of inducing luminal structure formation by culturing vascular endothelial cells in a collagen gel approximating physiological pressure (approximately 0.3 kPa). Under these culture conditions, both LPA4 receptors and entry receptors for SARS-CoV-2 infection, including ACE2, TMPRSS2, and Nrp1, were expressed by endothelial cells, indicating a phenotype similar to that of vascular endothelial cells in vivo. Multiple studies have reported the presence of SARS-CoV-2 viral particles in vascular endothelial cells in the lungs of patients with COVID-19^3,29,30^, though debate remains about whether this represents productive infection or viral interaction without replication^9,10^. In addition, infection of cultured vascular endothelial cells with SARS-CoV-2 has been reported to disrupt vascular endothelial cell junctions^31^. Studies suggest that vascular damage may primarily result from inflammatory cytokine effects rather than direct viral infection^6,32–34^ highlighting the complexity of vascular pathology in COVID-19. The evaluation system employed in this study, using 3D cultures of vascular endothelial cells, facilitated the analysis of the luminal vessel structure. Moreover, it also allowed for a closer approximation of SARS-CoV-2 infection to that observed in vivo. The experiments using these 3D culture systems indicated that SARS-CoV-2 infection in endothelial cells results in the upregulation of several key pro-inflammatory signaling pathways, while LPA treatment effectively downregulates these pro-inflammatory pathways. In addition, our animal studies on infected Syrian hamsters showed that LPA protects and stabilizes blood vessels and suppresses the pulmonary inflammatory characteristics of COVID-19. Our pathway analysis identified significant transcriptional changes in inflammatory signaling pathways, though future studies should validate these findings at the protein level through western blot analyses of key signaling molecules. Based on the findings in this study, the activation of LPA signaling in vascular endothelial cells is expected to represent an effective therapeutic approach for mitigating vascular injury in COVID-19.

Additional therapeutic agents targeting blood vessels may also be effective against new mutant strains of SARS-CoV-2 and other emerging viruses. To prepare for future pandemics that may occur at any time, it is important to develop a vasoprotective drug that targets LPA as an immediate treatment until a vaccine is developed. Our previous work strongly implicated LPA4 as the primary receptor involved in the vasoprotective effect of LPA against SARS-CoV-2^18^. However, LPA has been shown to exert various physiological effects through multiple receptors, and LPA administration may lead to effects other than vascular protection. Therefore, experiments using selective LPA4 agonists are needed to establish its role in protecting against SARS-CoV-2-induced vascular damage. Moreover, the relative contributions of different LPA receptors, including LPA4, to the vasoprotective effects we observed warrant further investigation. Translating these findings into clinical practice will require pharmacokinetic studies and safety trials with selective LPA4 agonists, followed by controlled clinical studies in patients with COVID-19 and early vascular dysfunction.

Despite the important findings of this study, there are certain limitations worth noting. First, while our 3D culture system provides valuable insights into vascular responses to SARS-CoV-2, it lacks the complex interactions between different cell types present in vivo, including pericytes and other supporting cells that contribute to vascular barrier function. Second, our animal model focused primarily on the pulmonary effects of SARS-CoV-2 infection, but COVID-19 is known to affect multiple organ systems. Future studies should explore the effects of LPA on vascular damage in other tissues affected by COVID-19. Finally, additional research is needed to understand the potential side effects of LPA treatment and to develop more targeted approaches, such as selective LPA4 receptor agonists, to maximize therapeutic benefits while minimizing adverse effects.

## Methods

### Viruses

Various SARS-CoV-2 strains, hCoV-19/Japan/QHN002/2021 (Alpha), hCoV-19/Japan/TY8-612/2021 (Beta), hCoV-19/Japan/TY7-503/2021 (Gamma), hCoV-19/Japan/TY11-927/2021 (Delta), and 2019-nCoV/Japan/TY38-873/2021 (Omicron), isolated at the National Institute of Infectious Diseases (Japan), were used in this study. We primarily used the Delta variant for our experiments because it was the predominant circulating strain during our study and has been associated with severe vascular complications^35^. SARS-CoV-2 was propagated in VeroE6/TMPRSS2 cells. The virus stock was generated from the supernatant of VeroE6/TMPRSS2 cells infected with SARS-CoV-2 at a multiplicity of infection of 0.1 and harvested 2 days after infection. The viral titer was determined using a plaque assay.

### Plaque formation assay

Vero/TMPRSS2 (JCRB1818) was obtained from JCRB Cell Bank in National Institutes of Biomedical Innovation, Health and Nutrition. VeroE6/TMPRSS2 cells were seeded into 24-well plates (80,000 cells/well) at 37 °C in 5% CO_2_ overnight. The supernatants were serially diluted using the inoculated medium and incubated for 2 h. Next, the culture medium was removed, fresh medium containing 1% methylcellulose (1.5 mL) was added, and the cells were cultured for an additional 3 days. Finally, the cells were fixed with 4% paraformaldehyde in PBS (Nacalai Tesque, Inc. Kyoto, Japan), and the plaques were visualized using Giemsa’s azur-eosin-methylene blue solution (#109204, Merck Millipore, Darmstadt, Germany).

### Reagents

The 1-oleoyl-LPA was purchased from Avanti Polar Lipids (Alabaster, AL, USA). LPA stock solution (10 mM) was prepared using 50% ethanol and stored at − 20 °C before use. LPA working solution was prepared by diluting the 50% stock solution in PBS at a ratio of 18:1. LPA (10 mg/mL) was used for study. For control experiments, 5% ethanol was used as the vehicle.

### Cell culture

Human Coronary Artery Endothelial Cell (HCAEC), HMVEC-Lung, and Human Pulmonary Artery Endothelial Cell (HPAEC) lines were purchased from Lonza (Basel, Switzerland) and grown in EBM-2 supplemented with EGM-2 MV BulletKit. Human Cardiac Microvascular Endothelial Cell (HCMEC), Human Hepatic Sinusoidal Endothelial Cell (HHSEC), HRGEC, and HMBEC lines were purchased from ScienCell (Berlin, Germany) and grown in an endothelial cell medium containing FBS and endothelial cell growth supplement. The Human Saphenous Vein Endothelial Cell (HSaVEC) line was purchased from PromoCell (Heidelberg, Germany) and grown in an endothelial cell growth medium-2. Human vascular cells were maintained under conditions of 5% CO_2_ and 37 °C, below 80% confluency, and used in experiments at passage 7 or 8.

### Endothelial cell 3D culture model

All eight human endothelial cell lines described above were cultured in collagen gels with 3D embedding and evaluated for their ability to form 3D luminal structures. Detailed characterization was performed on the three selected cell types (HMVEC-Lung, HMBEC, and HRGEC) based on receptor expression profiles. Cellmatrix Type I-A (Nitta Gelatin, Osaka, Japan) was diluted to 0.15% with dilute hydrochloric acid (Fujifilm Wako Pure Chemical Corporation, Osaka, Japan) at pH 3.0 and reconstituted according to the manufacturer’s protocol. The reconstituted collagen gel was mixed with cultured vascular endothelial cells at a concentration of 5 × 10^5^ cells/150 µl. The collagen cell mixture solution was cultured at 37 °C in a CO_2_ incubator for 30 min in a glass-bottomed dish (Matsunami D141400, Osaka, Japan). After the gel hardened, 10 ng/ml of VEGF-A (PeproTech, Rocky Hill, NJ, USA) containing the culture medium was added. Following incubation in a humidified incubator at 37 °C and 5% CO_2_ for 2 days, CD31 staining was performed to identify luminal structures. Vascular endothelial cells cultured under 3D conditions were pretreated with 10 µM of LPA for 30 min, infected with SARS-CoV-2 (Wuhan strain and Delta strain) at a multiplicity of infection of 0.1, and incubated with 10 µM of LPA. Two days after infection, the 3D tubes were fixed with paraformaldehyde and stained with Alexa Fluor 488 anti-human CD31 antibody (BioLegend, San Diego, CA, USA). Images were obtained using a TCS SP8 confocal microscope (Leica Microsystems, Wetzlar, Germany). The 3D tube volume was analyzed using Volocity imaging software (Perkin Elmer, Waltham, MA, USA). The weakest 10% of Alexa Fluor 488 fluorescence signals were excluded from the analysis as gel autofluorescence signal noise. Furthermore, objects smaller than 500 µm^3^ were excluded from the volumetric analysis as endothelial cells that were not 3D tube structures.

### Animals

Four-week-old male Syrian hamsters were purchased from Japan SLC (Shizuoka, Japan). The animals were housed in environmentally controlled rooms at the animal experimentation facility of Osaka University under standard conditions (22 ± 2 °C, 12 h light/dark cycle, standard laboratory diet, and water *ad libitum*). Animals were maintained in individually ventilated cages (ISOrat900 N, Tecniplast, Buguggiate, Italy) with appropriate bedding, enrichment, and air filtration. All experiments were performed according to the guidelines of the Osaka University Committee for Animal and Recombinant DNA Experiments (approval number 4062).

### Animal infection experiments

Syrian hamsters were randomly assigned to experimental groups (*n* = 4 animals per group: mock infection control, SARS-CoV-2 infection, SARS-CoV-2 infection + LPA 10 mg/kg, and SARS-CoV-2 infection + LPA 30 mg/kg) following established protocols from our previous work^20,21^. Syrian hamsters were anesthetized with isoflurane and challenged with 1.0 × 10^6^ PFU (in 60 µL) SARS-CoV-2 via intranasal routes. Infection was confirmed by the development of consistent respiratory symptoms and characteristic pulmonary pathology in SARS-CoV-2-inoculated animals, which were absent in mock-infected controls. To evaluate the therapeutic effects of LPA on vascular injury caused by SARS-CoV-2 infection, LPA (10 or 30 mg/kg, intraperitoneally) was administered daily from days 2 to 4 after infection. The selection of doses (10 and 30 mg/kg) was based on our previous studies with LPA in other mouse models^18^ and the treatment window (days 2–4) was chosen to reflect a clinically relevant scenario where treatment would begin after symptom onset. Five days post-infection, the hamsters were euthanized by isoflurane overdose followed by cervical dislocation, and the lungs were harvested for the experiments. All animal experiments with SARS-CoV-2 were performed at the Animal Biosafety Level 3 (ABSL3) facilities of the Research Institute for Microbial Diseases at Osaka University. Animal Experimentation, and the study protocol was approved by the Institutional Committee of Laboratory Animal Experimentation of the Research Institute for Microbial Diseases, Osaka University (approval number R02-08-0). All methods were performed in accordance with the relevant guidelines and regulations. All animal experiments were conducted in strict accordance with the guidelines for the care and use of laboratory animals established by the Ministry of Education, Culture, Sports, Science and Technology of Japan, and followed the ARRIVE guidelines for reporting animal research.

### RNA isolation and expression analysis

Vascular endothelial cells cultured in planar or 3D cultures were collected, and RNA was isolated using the RNeasy Mini Kit (Qiagen, Hilden, Germany) according to the manufacturer’s protocol. Total RNA was extracted from the lung tissue using TRIzol reagent. RNA was transcribed into cDNA using the PrimeScript RT Reagent Kit (TaKaRa, Shiga, Japan) according to the manufacturer’s instructions. Quantitative real-time PCR was performed on a LightCycler 96 (Roche, Rotkreuz, Switzerland) using the TB Green™ Premix Ex Taq™ Kit (TaKaRa) and was run in triplicate. The results were normalized to glyceraldehyde-3-phosphate dehydrogenase (GAPDH) levels using the comparative threshold cycle method. Specific primers used in this experiment were as follows: LPA4: 5’-CCTAGTCCTCAGTGGCGGTA-3’ (sense) and 5’-CTTCAAAGCAGGTGGTGGTT-3’ (anti-sense); GAPDH: 5’-GAAGGTGAAGGTCGGAGTC-3’ (sense) and 5’-GAAGATGGTGATGGGATTTC-3’ (anti-sense); ACE2: 5’-GGATTCCATGAAGCTGTTGG-3’ (sense) and 5’-TCGTGAGTGCTTGTTTGAGC-3’ (anti-sense); and TMPRSS2: 5’-TGGAGCCGGATACCAAGTAG-3’ (sense) and 5’-GTTGGGCAGACACACTGGTT-3’ (anti-sense). Three independent samples were collected from each group, and mRNA was extracted from endothelial cells using RNeasy Plus Micro Kits (Qiagen) according to the manufacturer’s protocol. RNA libraries were prepared using the TruSeq Sample Prep v2 kit and sequenced on a HiSeq 2500 (Illumina, San Diego, CA, USA) in the 75-base single-end mode. CASAVA 1.8.2 software (Illumina) was used for base calling. Sequenced reads were mapped to the mouse reference genome sequence (mm9) using TopHat v2.1.0. Fragments per kilobase of exons per million mapped fragments values were determined using Cuffnorm v.2.2.1.

### Tissue staining

Lung tissue samples harvested from hamsters were fixed in 4% paraformaldehyde, and cryo-tissue sections were prepared. Hematoxylin and eosin staining was performed to evaluate immune cell infiltration and pathological changes in lung tissues, with 20 random high-power fields (400× total magnification) scored for each condition. Scores were assigned to each parameter, including neutrophil infiltration in the alveolar and interstitial spaces, hyaline membrane, proteinaceous debris filling the airspaces, and alveolar septal thickening^36^. Histopathological evaluation was performed by a pulmonologist who assessed the extent of inflammatory lesions and vascular damage in a blinded manner using a standardized scoring system. The blood vessels of the lung sections were stained with *Lycopersicon esculentum* Lectin, DyLight 488 conjugate (Vector Laboratories, Burlingame, CA, USA), and TO-PRO-3 stain (ThermoFisher, Waltham, MA, USA). Tissue preparation and staining were performed as previously described^37^.

### DEG analysis

The RNA-seq data in Fig. 3 were analyzed using iDEP (ver2.01) (http://bioinformatics.sdstate.edu/idep/)^38,39^. DEGs were identified using DESeq^40^ with a maximum FDR p-value of < 0.05 and a fold change of at least 1.5 between groups. We performed k-means clustering^40^ on the 2,000 most variable genes using the Gene Ontology biological process^41^ and KEGG pathway databases^42^. We analyzed the RNA-seq data (Fig. 5) obtained under the three experimental conditions to identify genes with differential expression patterns. The conditions were as follows: (1) Samples collected 5 days after SARS-CoV-2 infection (SARS-CoV-2); (2) samples collected 5 days after SARS-CoV-2 infection with subsequent LPA stimulation on days 2, 3, and 4 post-infection (SARS-CoV-2 + LPA); and (3) samples collected 5 days after mock SARS-CoV-2 infection served as controls (mock). Four samples were analyzed for each condition. We then extracted genes exhibiting expression differences under the following conditions: (1) Genes with increased expression post-infection compared to non-infection (SARS-CoV-2) were selected if the log2 ratio of the average gene expression in SARS-CoV-2-infected samples to that in non-infected samples was ≥ log2(1.5), and the average expression in SARS-CoV-2-infected samples was at least 1.0. (2) Genes with decreased expression under LPA stimulation compared to SARS-CoV-2 infection (SARS-CoV-2 + LPA) were selected if the log2 ratio of the average gene expression in SARS-CoV-2 + LPA samples to that in SARS-CoV-2-infected samples was ≤ log2(0.75), and the average expression in SARS-CoV-2-infected samples was at least 1.0. Using these criteria, we identified specific gene sets that exhibited altered expression in response to SARS-CoV-2 infection and subsequent LPA stimulation.

### Gene enrichment analysis

To explore the functional characteristics of the genes altered by SARS-CoV-2 infection, we performed gene enrichment analysis on two groups: those with increased expression following SARS-CoV-2 infection and those exhibiting decreased expression upon LPA stimulation. Fisher’s exact test was used to identify functionally enriched gene groups using Gene Ontology sets from MsigDB (c5.all.v7.2. symbols.gmt). This approach delineates the functional pathways and biological processes involved in these gene sets.

### Statistical analysis

All data are presented as mean ± SD. No data were excluded from the analyses. For the in vivo studies, age-matched and weight-matched hamsters were randomized into experimental or control groups. Statistical analysis was performed using the statcel4 software package (OMS, Saitama, Japan) with analysis of variance for all data, followed by the Tukey–Kramer multiple comparisons test. Two-sided Student’s t-tests were used to compare two groups. *P* < 0.05 was considered to indicate statistical significance.

## Electronic supplementary material

Below is the link to the electronic supplementary material.

Supplementary Material 1

## Data Availability

The datasets generated and analyzed in this study are available from the corresponding author upon request. The RNA-seq data used in this study are available from GEO under the accession number PRJDB18309 (https://ddbj.nig.ac.jp/search/entry/bioproject/PRJDB18309).
