# Supplementary material for: Vasoprotective effects of lysophosphatidic acid inhibit vascular injury caused by SARS-CoV-2 infection
Source: Sci Rep. 2025 Jul 25;15:24622. doi: 10.1038/s41598-025-06569-7 (PMC12297521; doi:10.1038/s41598-025-06569-7)
Supplement: Supplementary file 1 — Supplementary Material 1 [file 41598_2025_6569_MOESM1_ESM.pdf]

**Vasoprotective effects of lysophosphatidic acid inhibit vascular injury caused by SARS-CoV-2 infection**

Fumitaka Muramatsu, Naoi Hosoe, Tatsuya Suzuki, Teppei Shimamura, Yumiko Hayashi, Kazuhiro Takara, Lamri Lynda, Anna Shimizu, Weizhen Jia, Yoshimi Noda, Nobuyuki Takakura, Toru Okamoto, and Hiroyasu Kidoya

**Contact:** Hiroyasu Kidoya, *Department of Integrative Vascular Biology, Faculty of Medical Sciences, University of Fukui, 23-3 Matsuoka-Shimoaizuki, Eiheiji, Yoshida, Fukui 910-1193, Japan*; Phone: +81-776-61-8286; E-mail: kidoya@u-fukui.ac.jp

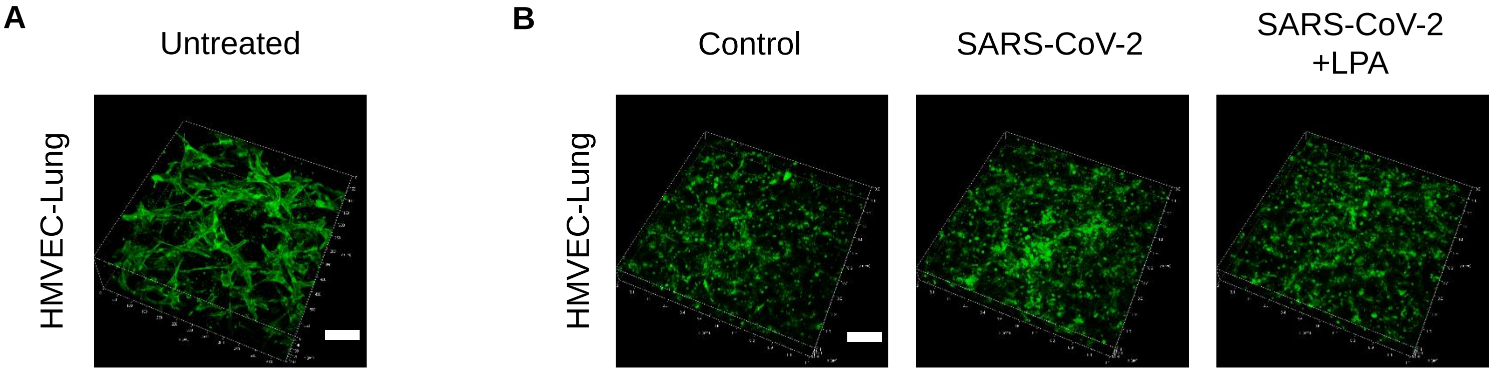


**Supplemental Figure 1. Analysis of the vascular structure in 3D-cultured HMVEC-Lung**

**(A)** CD31-immunostaining of 3D-cultured HMVEC-Lung without viral infection. Images were obtained from the bottom of the culture dish to 160 μm. Scale bar, 100 μm

**(B)** Representative image data of CD31-immunostaining of 3D-cultured HMVEC-Lung infected with SARS-CoV-2 and treated with LPA. Images were obtained from the bottom of the culture dish to 100 μm. Scale bar, 100 μm

3D: three-dimensional; HMVEC: microvascular endothelial cells; SARS-CoV-2: severe acute respiratory syndrome-related coronavirus-2; LPA: lysophosphatidic acid
